# Public Education and Electronic Awareness of the New Coronavirus (COVID-19): Experiences From Iran

**DOI:** 10.1017/dmp.2020.94

**Published:** 2020-04-14

**Authors:** Mahmoudreza Peyravi, Milad Ahmadi Marzaleh, Navvab Shamspour, Ahmad Soltani

**Affiliations:** Assistant Professor, Department of Health in Disasters and Emergencies, Health Human Resources Research Center, School of Management and Medical Informatics, Shiraz University of Medical Sciences, Shiraz, Iran; Research Center for Emergency and Disaster Resilience, Red Crescent society of the Islamic Republic of Iran, Tehran, Iran; Health Policy, Health Policy Research Center, Institute of Health, Shiraz University of Medical Sciences, Fars, Iran; Research Center for Health Management in Mass Gathering, Red Crescent society of the Islamic Republic of Iran, Tehran, Iran; Islamic Azad University, Tehran North Branch, Tehran, Iran

The first case of the new Coronavirus (COVID-19) with a high transmission was identified in December 2019 in Wuhan, China. COVID-19 is highly contagious, and the World Health Organization (WHO) declared a global health emergency on 30 January 2020.^1-3^ Human-to-human transmission, human population movement, and climatic conditions can transmit this virus faster. The most common clinical symptoms of the disease include muscle cramps, fever, cough, fatigue, and headaches.^4,5^ Approximately 20% of the affected patients showed severe symptoms, and the reported rate of mortality was approximately 3%.^6^ The first positive confirmed case of the disease in Iran was announced on February 18, 2020, and new cases were identified in all cities in Iran. When WHO declared a global health emergency, the Iranian Red Crescent Society and Ministry of Health took measures for public awareness. Following the outbreak of the coronavirus, and due to the necessity of notification, especially in health issues and personal hygiene, the following steps have been taken by the Iranian organizations:

1. Conducting a research workshop to prepare, translate, and compile scientific content needed by the public on prevention issues;
2. Holding training and planning workshops to develop materials according to the audience;
3. Holding station training in public places to show how to wash the hands ;
4. Producing interconnected info-graphs to provide awareness about how to prevent the disease, wash the hands, properly use a mask, strengthen the immune system, deal with a suspected infected person, deal with stress from illness, and to react to the rumors. More than 20 million hits, views, and likes on social media including Telegram, Instagram, and Twitter reported;
5. Distributing and installing public awareness posters in complexes and apartment buildings;
6. Holding e-training courses for the general public;
7. Sending mass short messages by means of mobile phones to all people;
8. Producing infographs for Arab and Turkish citizens in neighboring countries;
9. Launching the *corona.ir* website in collaboration with the medical system to load the content needed by different groups with millions of views;
l0. Creating a special group of reporters on crisis and educational information to be broadcast in mass media;
11. Making different groups for providing information and notifications on social networks;
12. Developing educational materials for the deaf and target groups such as children and drivers in partnership with banks and the Islamic Republic of Iran Medical Council;
13. Developing content for banking users.

The process of developing content with a focus on specific groups is continuing.

An overview of 20,000 comments by the learners of the e-training showed 86% satisfaction by the people and high effectiveness (79%) by the participants. Sixty-nine percent of the participants announced the trainings helped them reduce the disease-borne stress and how to mitigate the virus’s impacts. Comparing the online training methodology with ordinary ones, 82% of the participants declared the first is safer with regard to the disease outbreak. The participants asked for a question and answer section, which was added as off-line access later on. Following the outbreak in China and later in Iran, the infographic contents circulation indicates exponential growth in sharing of the materials among the public on the Web, social media, and popular applications. The comments by users indicated 86% satisfaction. Some comments (12%) expressed the infographics are autonomously placed in apartments, companies, and organizations. The feedback from the public expressed that those materials, including videos containing humor, animation, and kid-friendly themes, are viewed more and had possibly higher effectiveness than those with a monolog lecture format. These contents were more attractive to different genders and age groups.

Focus on special public groups, such as drivers and bakeries, and observing the hygienic principles in social activities, such as shopping, haircutting, and gas stations, are considered more successful among people of different walks of life than are some general advice formats. Using disinfectants and masks were among the training challenges containing questions and contradictions. Paying attention to the social vulnerabilities, livelihoods, and post-Corona social, psychological, and economic crises are the most challenging concerns that must be later taken into consideration. The new approach to the e-training and virtual contexts was an opportunity for the public awareness system in Iran followed by solemn attention to training.

## CONCLUSION

Training and education of preventive measures and how to deal with infection and exposure to infected patients are the most important steps to cope with COVID-19. Timely risk identification and designing an educational protocol regarding the widespread outbreak of the virus, and provision of proactive, timely, and effective public education should be considered as well. The widespread acceptance and rapid dissemination of messages generated in cyberspace and a high number of visitors reflect the effective and timely use of this space to solve these health problems. However, new educational technologies and applications and the capacity of national and private media should also be taken into account. Simple, functional, and free online courses should be available to everyone; user-friendly for the illiterate and disabled should also be considered. In addition, the comments by users and visitors on modifying and updating training messages increase the effectiveness of the training method. The educational content in different media formats can be developed and dedicated to the age of the target audience to enhance learning effectiveness. Training enhances the people’s proper readiness and response to the virus; so, to manage the virus, policy-makers in the health system and all organizations involved should take steps in providing innovative, unified, and applied educational content to all people. It is also important and socially responsible for organizations and the people in these educational contexts to promote community resiliency.
